# Global Push: Multicontinent Project Assesses Particulate Matter and Birth Weight

**DOI:** 10.1289/ehp.121-a94

**Published:** 2013-03-01

**Authors:** Tanya Tillett

**Affiliations:** Tanya Tillett, MA, of Durham, NC, is a staff writer/editor for *EHP*. She has been on the *EHP* staff since 2000 and has represented the journal at national and international conferences.

Maternal exposure to ambient air pollution has been associated with a number of adverse pregnancy outcomes including low birth weight, defined as a full-term infant weighing less than 2,500 g (about 5 lb, 8 oz) at birth. But it’s been difficult to translate these findings into health-protective policies because of inconsistencies across studies—something the International Collaboration on Air Pollution and Pregnancy Outcomes (ICAPPO) is working to remedy. This worldwide, multicenter project used a common analysis protocol to derive combined effect estimates of maternal exposure to coarse and fine particulate matter (PM_10_ and PM_2.5_, respectively) and to assess differences in how the centers conducted their individual studies [*EHP 121(3):367–373; Dadvand et al.*].

ICAPPO researchers at 14 centers in 9 countries generated effect estimates for ambient PM_10_ and PM_2.5_ in relation to more than 3 million live, singleton, term births. For this analysis, the authors used a meta-analysis approach to synthesize contributing factors such as study setting, availability of air pollutant data, birth data, and maternal socioeconomic status. They estimated combined effects across centers and used meta-regression analysis to determine how each center’s particular characteristics and exposure assessment methods influenced differences in effect estimates among centers. All these factors can contribute to variation in results across studies.

Higher maternal exposure to PM_10_ and PM_2.5_ was associated with lower birth weight across the study population. The investigators found a positive association between low birth weight and increased PM_10_ and PM_2.5_ exposure during the entire pregnancy, as well as a negative association between increased PM_10_ exposure and term birth weight when all averages were compared.

**Figure:**
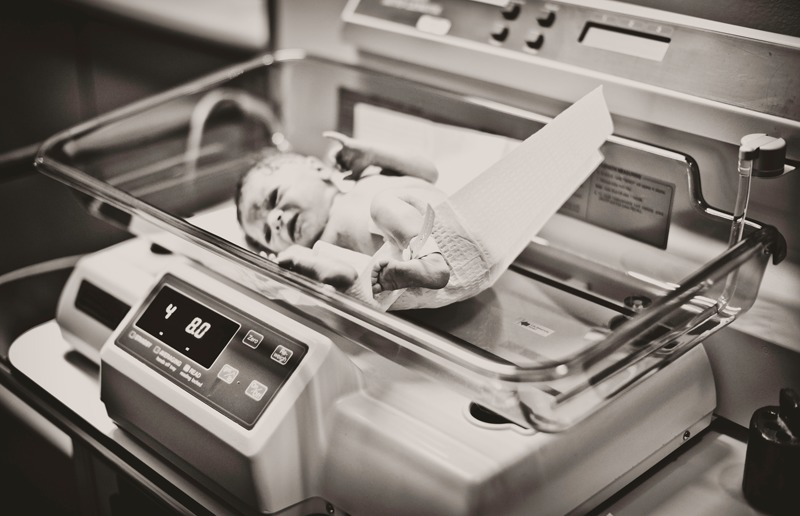
Analysis of more than 3 million births worldwide yielded strong evidence of a link between low birth weight and prenatal exposure to particulate matter air pollution. © Kangah/iStockphoto.com

Differences in average PM_2.5_ exposure levels and PM_2.5_/PM_10_ ratios influenced effect estimate outcomes—centers with higher PM_2.5_ levels and higher PM_2.5_/PM_10_ ratios reported stronger associations with birth weight. These findings could indicate a geographical variation in the association between air pollution and low birth weight. Meta-regression analyses also showed that centers reported stronger associations if they conducted a temporal exposure assessment only (i.e., exposure was determined according to the time period that each woman was pregnant) compared with those that accounted for both spatial and temporal elements of exposure (i.e., exposure was determined by time of pregnancy and where the woman lived).

Although the study does not include direct measurements of personal exposure levels, it does provide a comprehensive estimate of the global effects of maternal exposure to particulate matter on birth weight. This could prove useful for shaping meaningful public health policies regarding air pollution.

